# Addition of buttermilk powder improved the rheological and storage properties of low‐fat yogurt

**DOI:** 10.1002/fsn3.1373

**Published:** 2020-05-27

**Authors:** Lili Zhao, Ran Feng, Xueying Mao

**Affiliations:** ^1^ Beijing Advanced Innovation Center for Food Nutrition and Human Health College of Food Science & Nutritional Engineering China Agricultural University Beijing China; ^2^ Key Laboratory of Functional Dairy Ministry of Education College of Food Science and Nutritional Engineering China Agricultural University Beijing China; ^3^ College of Food Science and Engineering Northwest A&F University Yangling China

**Keywords:** buttermilk, low‐fat yogurt, rheological properties, storage properties

## Abstract

Buttermilk is used widely in dairy products due to its good emulsifying and nutritional properties. In the present study, 0%–4.0% (w/w) buttermilk powder was added to low‐fat yogurt with a constant protein content to investigate its efficacy on the rheological and storage properties of low‐fat yogurt. Buttermilk increased the final titration acidity. Addition of buttermilk decreased the pH at the gelation point, shortened the gelation time, and thus shortened the fermentation period. Storage modulus G', yield stress, yield strain, and compact cross‐links of the microstructure were enhanced greatly with addition of 1.0%‐2.0% (w/w) buttermilk powder. In addition, addition of buttermilk decreased whey separation and increased the viscosity and firmness of low‐fat yogurt during storage. Our findings suggest that the addition of an appropriate amount of buttermilk altered the rheological characteristics and improved the textural and storage properties of low‐fat yogurt.

## Graphical abstract

In present study, 0%–4.0% (w/w) buttermilk powder was added to low‐fat yogurt with a constant protein content to investigate its efficacy on the rheological and storage properties of low‐fat yogurt. Addition of 1.0%‐2.0% (w/w) buttermilk powder could accelerate the fermentation period, decrease whey separation, and improve rheological, textural, and storage properties.

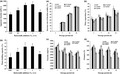

## 1 INTRODUCTION

Yogurt is a widely consumed fermented dairy food, because of its digestibility, nutritional value, and health benefits (Shah, 2013). In recent years, low‐fat yogurt has been attracting more attention from consumers, because fat is associated with an increased risk of obesity, coronary heart disease, and elevated blood pressure (Kaminarides, Stamou, & Massouras, 2007). However, fat reduction can cause some undesirable characteristics in low‐fat yogurt, including poor texture, low viscosity, high syneresis, and lack of flavor, especially during the storage period, which results ultimately in poor mouthfeel and may hinder consumer acceptance of low‐fat yogurt (Lee & Lucey, 2010). Therefore, to overcome the deficiencies in texture, mouthfeel, and physical properties of low‐fat yogurt, many methods have been explored, such as the addition of milk‐derived ingredients, thickeners, and stabilizers (Andiç, Boran, & Tunçtürk, 2013; Peng, Horne, & Lucey, 2009; Sodini, Remeuf, Haddad, & Corrieu, 2004). Viscous characteristics of low‐fat yogurt were also enhanced by the addition of exopolysaccharides (EPS)‐producing starter cultures, because of the interactions of EPS with the casein network (Vlahopoulou & Bell, 1993). An optimum level of transglutaminase, which established a covalent bond between glutamine and lysine residues in milk proteins, resulted in ideal texture and whey retention in pizza cheese (Gharibzahedi & Chronakis, 2017). In order to manufacture clean‐label yogurts without additives, addition ingredients from milk itself (e.g., nonfat dry milk, whey proteins, sodium caseinate) or adjustment of the ratio of casein to whey protein in milk have been used to obtain ideal texture of yogurts (Isleten & Karagul‐Yuceer, 2006; Zhao, Wang, Tian, & Mao, 2016). But these methods were less focused on improving the nutrition of low‐fat yogurt. Buttermilk possesses a variety of nutritional advantages. Buttermilk is a source of milk fat globule membranes that carry many health benefits, including inhibition of colon cancer, suppression of gastrointestinal pathogens, and alleviation of stress responses (Spitsberg, 2005). Buttermilk may satisfy consumer demand for both texture and nutrition in low‐fat yogurt.

Buttermilk is a by‐product from the manufacture of butter and anhydrous milk fat. It contains the water soluble components of milk, which include casein, whey proteins, lactose, and a higher proportion of milk fat globule membranes (Roesch, Rincon, & Corredig, 2004). Casein, whey proteins, and milk fat globule membranes in buttermilk possess inherent emulsifying properties. Buttermilk has been used in bakery, chocolate, cheese, yogurt, and for the delivery of bioactives as wall material for encapsulation (Augustin et al., 2015; Govindasamy‐Lucey, Lin, Jaeggi, Johnson, & Lucey, 2006; Le et al., 2011; Morin, Pouliot, & Britten, 2008; Romeih, Moe, & Skeie, 2012; Trachoo & Mistry, 1998). Buttermilk improved crumb texture (Madenci & Bilgiçli, 2014), enhanced water‐holding capabilities of yogurt (Le et al., 2011; Romeih, Abdel‐Hamid, & Awad, 2014), increased yield, prolonged the shelf life of cheese (El Sayed et al., 2010), and increased in vitro bioaccessibility of bioactives (Augustin et al., 2015). Therefore, good emulsification, water‐holding capacity, and desirable nutritional properties make buttermilk a good choice for improving the nutrition and texture of low‐fat yogurt.

Although the effect of buttermilk on skim yogurt has been investigated, the results from different investigations were different. Le et al. (2011) found that the addition of buttermilk did not improve the texture of skim yogurt with constant dry solid content, but Romeih et al. (2014) found that buffalo fat‐free yogurt with addition of buttermilk had a more compact and a denser gel structure without controlling the dry solid. Therefore, whether addition of buttermilk is able to improve the texture of low‐fat yogurt needs further investigation. In addition, little information is available on the effect of buttermilk fortification on the rheological properties of low‐fat yogurt during fermentation and structural attributes of low‐fat yogurt during storage. Poor storage properties have a negative impact on the shelf life of low‐fat yogurt (Srisuvor, Chinprahast, Prakitchaiwattana, & Subhimaros, 2013). Therefore, the main objective of our study was to investigate the effect of buttermilk on the fermentation and storage properties of low‐fat yogurt under a constant level of protein. We quantified the changes in acidity and rheological properties during fermentation of low‐fat yogurt that was fortified with different concentrations of buttermilk. The changes in texture and postacidification of buttermilk‐enriched low‐fat yogurt during storage were also evaluated to clarify the optimal amount of buttermilk powder to use as an additive.

## 2 MATERIALS AND METHODS

### 2.1 Chemicals and materials

Skim milk powder (SMP) and buttermilk powder (BMP) were purchased from Fonterra Cooperative Group Ltd. Direct Vat Set (starter culture Yo‐C798‐F) that contained *Streptococcus thermophiles* and *L. delbrueckii subsp. bulgaricus* was obtained from Inatural Biological Technology Co., Ltd. Other reagents used in the present study were of analytical grade.

From the supplier information, BMP contained 31.0% protein, 7.8% fat, 1.3% phospholipid, 3.8% moisture, 50.0% lactose, and 7.4% minerals. SMP contained 34.0% protein, 1.9% fat, 2.6% moisture, 52.1% lactose, and 9.4% minerals.

### 2.2 Milk base and yogurt preparation

#### 2.2.1 Milk base preparation

Five different milk bases were produced. Each total milk base was 500 g and protein content of all samples was 4.0% (w/w). Low‐fat milk base without buttermilk was used as the control and was made with 11.8% solids from SMP. The other four different low‐fat milk bases were made with 11.3%, 10.9%, 9.9%, and 8.1% solids from SMP, into which 0.5%, 1.0%, 2.0%, and 4.0% solids from BMP were added, respectively, as shown in Table 1. The milk bases were stirred for 2 hr at room temperature and stored at 4°C overnight until completely hydrated.

**Table 1:** The formula of milk base with different concentrations of buttermilk powder

	0% BMP	Addition of buttermilk			
		0.5% BMP	1% BMP	2% BMP	4% BMP
SMP (g/100 g)	11.8	11.3	10.9	9.9	8.1
BMP (g/100 g)	0	0.5	1.0	2.0	4.0
Total protein (%)	4.0	4.0	4.0	4.0	4.0
Total solids (%)	11.8	11.8	11.9	11.9	12.1

Note

0% BMP, 0.5% BMP, 1% BMP, 2% BMP, and 4% BMP mean low‐fat yogurts with 0%, 0.5%, 1%, 2%, and 4% added buttermilk powder, respectively.

Abbreviations: BMPbuttermilk powder; SMP, skim milk powder.

#### 2.2.2 Yogurt preparation

Five hundred grams of each milk base was heated in a water bath (DK‐8B, Jing Hong Test Equipment Co., LTD) until the core temperature of samples reached 95°C, where it was held for 5 min. After cooling to 42°C, the direct Vat Set was added at a recommended concentration of 0.01% (w/w). Each milk base was separately loaded in beakers for the subsequent experiment. All samples were fermented at 42°C for 4 hr. All samples were prepared in triplicate. Major nutrients in the yogurt samples were determined (Table 2).

**Table 2:** Compositions of low‐fat yogurt and low‐fat yogurt with different concentrations of buttermilk powder

	Protein (%)	Fat (%)	Phospholipid (%)	Lactose (%)	Minerals (%)
Low‐fat yogurt	3.97 ± 0.32^a^	0.14 ± 0.02^d^	0.01 ± 0.00^e^	6.20 ± 0.63^a^	0.84 ± 0.10^a^
0.5% BMP	4.02 ± 0.13^a^	0.19 ± 0.01^c^	0.05 ± 0.01^d^	6.33 ± 0.77^a^	0.87 ± 0.13^a^
1% BMP	3.92 ± 0.85^a^	0.22 ± 0.03^c^	0.11 ± 0.01^c^	5.95 ± 0.57^a^	0.87 ± 0.11^a^
2% BMP	4.01 ± 0.44^a^	0.29 ± 0.02^b^	0.21 ± 0.01^b^	5.80 ± 0.89^a^	0.86 ± 0.03^a^
4% BMP	3.98 ± 0.45^a^	0.43 ± 0.04^a^	0.42 ± 0.02^a^	5.34 ± 0.33^a^	0.84 ± 0.02^a^

Note

^a‐e^Means with different letters within the same column are significantly different (*p* < .05).

0.5% BMP, 1% BMP, 2% BMP, and 4% BMP mean low‐fat yogurts with 0%, 0.5%, 1%, 2%, and 4% added buttermilk powder, respectively.

Abbreviations: BMP, buttermilk powder.

### 2.3 Determination of pH and titration acidity

The pH value and titration acidity were monitored every 1 hr during fermentation and every 7 day during the storage period. Titration acidity was measured according to the modified method described by Zhang, Zhao, Qu, Zhao, and Zhao (2010). The titration acidity during storage period was determined at 1, 7, 14, and 21 day. The increase rate of titration acidity during storage period was expressed as follows:

$$\text{Increase}\;\text{rate}\;\text{of}\;\text{titration}\;\text{acidity}\left(\%\right)=\left(\text{titration acidity at1}\;d\left(\text{or 7}\;\text{day}/14\;\text{day}/21\;\text{day}\right)-\text{titration}\;\text{acidity}\;\text{at}\;\text{1day}/\text{titration}\;\text{acidity}\;\text{at}\;1\;\text{day}\right)\times 100\%$$

### 2.4 Determination of firmness and apparent viscosity

Samples were fermented in beakers before measurement. Firmness was measured using a texture analyzer (TA‐XT21, Stable Micro System Company). The probe (SMS P/35) was penetrated to a depth of 10 mm at a speed of 2.0 mm·s^‐1^ (Awad, 2007). The samples were compressed to 50% of their original thickness in a double cycle. Firmess (N) was computed from the resulting force deformation curves.

The apparent viscosity was measured with a Brookfield digital rotational viscometer (SNB‐1, Precision Scientific Instrument Co., Ltd) using a spindle 4 at 12 rpm in 10 g yogurt. The spindle was allowed to rotate in the sample for 30 s at 15°C. The apparent viscosity was read in centipoise from the viscometer.

### 2.5 Determination of rheological properties

The rheological properties of low‐fat yogurt with or without added BMP during the fermentation process were monitored using a controlled‐strain rheometer (Physica MCR 301, Anton Paar Company) with low‐amplitude oscillation. Samples that were inoculated with starter culture were placed in a cylinder, and the temperature was maintained at 42°C. During fermentation, the oscillation mode was set with a frequency of 1 Hz and a constant strain of 0.1%. Measurements were taken every 5 min until pH 4.6 was reached. Storage modulus G', gelation time, and fermentation period were selected as descriptors of rheological properties. Gelation time was defined as the moment when the G' values of gels were greater than 1 Pa (Lucey, Munro, & Singh, 1999). Fermentation period was the time it took to reach a pH of 4.6.

The large deformation properties of yogurt gels were determined by applying a single constant shear rate (~0.01 s^‐1^). The strain that was applied varied from 0.01% to 100%. Yield stress (σ_yield_) was defined as the point when shear stress started to decrease, and yield strain (y_strain_) was the strain value at the yield point (Lucey, 2002).

### 2.6 Measurement of whey separation and water‐holding capacity

Yogurts were transferred into centrifuge tubes and centrifuged at 1,500 × *g* for 10 min at 4°C. Whey separation and water‐holding capacity were carried out according to the method of Isanga and Zhang (2009). The supernatant was taken out immediately, and its weight was recorded.

The whey separation was expressed as follows:

$$\text{Whey}\;\text{separation}\;\left(\%\right)=\left(\text{weight}\;\text{of}\;\text{supernatant}/\text{weight}\;\text{of}\;\text{sample}\right)\times 100\%$$

The water‐holding capacity was expressed as follows:

$$\text{Water}-\text{holding capacity}\left(\%\right)=\left(\left(\text{weight of sample}-\text{weight of supernatant}\right)/\text{weight of sample}\right)\times 100\%$$

### 2.7 Measurement of microstructure

Confocal scanning laser microscopy (CSLM) was used to evaluate the microstructure of yogurts as reported by Zhao et al. (2016), with few modifications. Rhodamine B (Sigma) at a concentration of 10 mg/ml was used to stain protein and *N*‐(Lissamine rhodamine B sulfonyl) dioleoylphosphatidyl ethanolamine (Rh‐DOPE, concentration of 1 mg/ml in chloroform; Avanti polar lipids Inc.) was used to label phospholipids. Two dyes and starter culture were added to 100 g of milk base and mixed with a magnetic stirrer (CJJ78‐1, Shanghai solid instrument co.) for approximately 5 min. A few drops of the sample were transferred to a concavity slide over which a coverslip was placed, which was then incubated at 42°C until the pH reached 4.6. The gel samples thus obtained were observed by a Leica TCS 4D confocal microscope (Leica Lasertechnik GmbH) with a 60 × oil immersion objective (numerical aperture = 1.4) at an excitation wavelength of 568 nm for Rhodamine B and 488 nm for Rh‐DOPE. The images had a resolution of 1,428 × 1,428 pixels, and the pixel scale values were converted into micrometers using a scaling factor.

### 2.8 Statistical analysis

All experiments were carried out three times. Data were expressed as means ± standard deviation (*SD*). Data were analyzed using one‐way ANOVA in SPSS software (version 13.0 for Windows, SPSS Inc.). Differences were considered significant at *p* < .05. Figures were produced using Origin 8 software (OriginLab Ltd.).

## 3 RESULTS

### 3.1 Changes in titration acidity and pH of low‐fat yogurt during fermentation

The changes in titration acidity and pH of yogurts with the addition of buttermilk were monitored during the fermentation process (Table 3). A decreasing trend in pH and an increasing trend in titration acidity were observed in all yogurt samples. Compared with low‐fat yogurt without buttermilk, a significantly higher titration acidity and a significantly lower pH were obtained for low‐fat yogurt with the addition of 2.0% and 4.0% (w/w) buttermilk at 4 hr of fermentation (*p* < .05). It demonstrated that the addition of 2.0% and 4.0% (w/w) buttermilk shortened the fermentation period of low‐fat yogurt.

**Table 3:** Changes of titration acidity and pH value of low‐fat yogurt with different concentrations of buttermilk powder during fermentation

	SMP	0.5% BMP	1% BMP	2% BMP	4% BMP
Titration acidity					
0 hr	13.75 ± 0.41^a^	14.00 ± 0.35^a^	14.50 ± 0.43^a^	14.25 ± 0.43^a^	15.00 ± 0.60^a^
1 hr	22.50 ± 1.23^a^	21.75 ± 0.54^a^	23.00 ± 0.69^a^	22.75 ± 0.68^a^	23.25 ± 0.93^a^
2 hr	42.63 ± 2.31^a^	42.25 ± 1.05^a^	44.00 ± 1.35^a^	42.75 ± 1.28^a^	42.88 ± 1.72^a^
3 hr	68.25 ± 2.05^a^	69.25 ± 1.73 ^a^	68.50 ± 2.06^a^	69.38 ± 2.08^a^	69.75 ± 2.79^a^
3.5 hr	75.63 ± 2.03^b^	75.75 ± 1.23 ^ab^	78.50 ± 2.36^ab^	80.75 ± 2.23^a^	80.00 ± 2.20^a^
4 hr	77.20 ± 1.54^c^	78.10 ± 1.95 ^bc^	80.00 ± 2.40^ac^	82.30 ± 2.47^a^	82.00 ± 1.28^a^
pH					
0 hr	6.64 ± 0.14^a^	6.60 ± 0.19^a^	6.54 ± 0.13^a^	6.53 ± 0.13^a^	6.50 ± 0.19^a^
1 hr	6.13 ± 0.12^a^	6.10 ± 0.18^a^	6.02 ± 0.12^a^	6.00 ± 0.12^a^	5.96 ± 0.18^a^
2 hr	5.69 ± 0.10^a^	5.59 ± 0.17^a^	5.57 ± 0.11^a^	5.55 ± 0.11^a^	5.54 ± 0.12^a^
3 hr	4.99 ± 0.15^a^	4.91 ± 0.15^a^	4.91 ± 0.09^a^	4.87 ± 0.09^a^	4.85 ± 0.15^a^
4 hr	4.64 ± 0.11^a^	4.59 ± 0.14^ab^	4.50 ± 0.09^ab^	4.45 ± 0.08^bc^	4.40 ± 0.09^bc^

Note

^a‐c^Means with different letters within the same row are significantly different (*p* < .05). 0.5% BMP, 1% BMP, 2% BMP, and 4% BMP means low‐fat yogurts with 0%, 0.5%, 1%, 2%, and 4% added buttermilk powder, respectively.

### 3.2 Rheological properties of low‐fat yogurts with the addition of buttermilk

**Figure 1:**
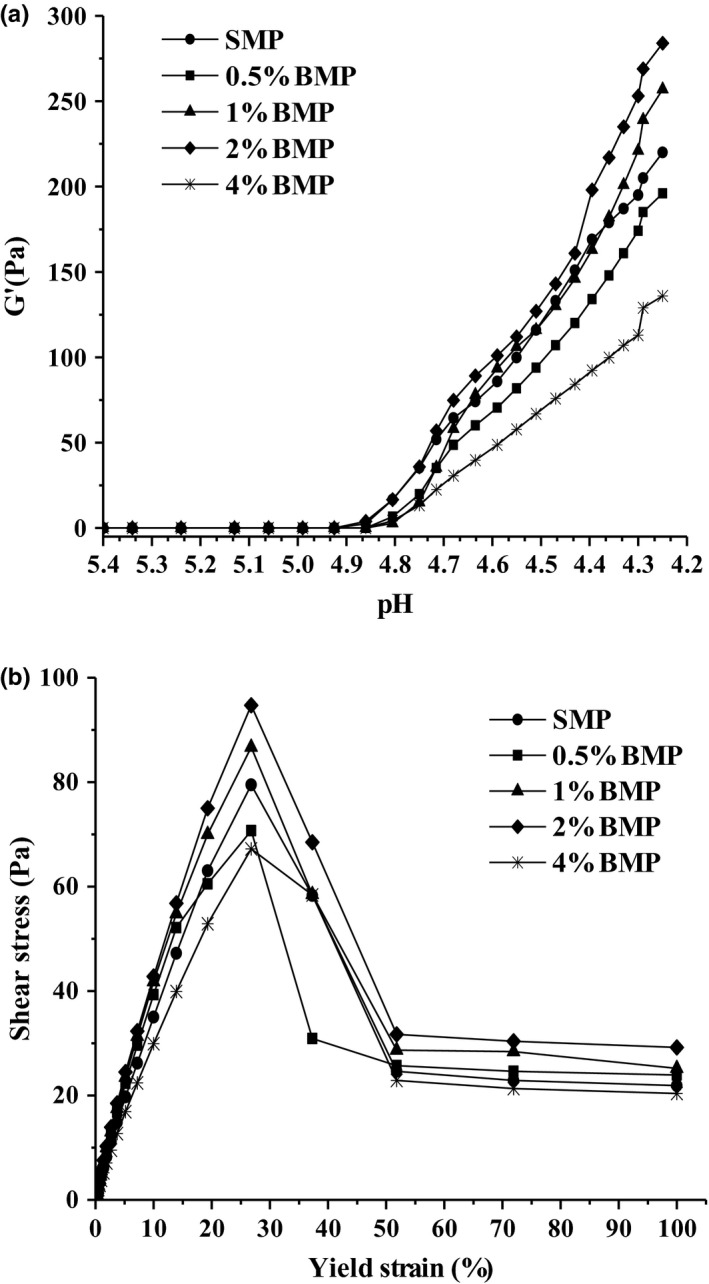
The change of storage modulus (G′) as a function of (a) pH and (b) shear stress as a function of yield strain for low‐fat yogurt with the addition of buttermilk powder. SMP, skim milk powder. BMP, buttermilk powder. 0.5% BMP, 1% BMP, 2% BMP, and 4% BMP mean low‐fat yogurts with 0%, 0.5%, 1%, 2%, and 4% added buttermilk powder, respectively

During the fermentation process, the increase rate of G' for yogurt with 2.0% buttermilk was higher than that of the other samples, which produced the highest final G' value (Figure 1a). The increase rate of G' for yogurt with 4.0% buttermilk was the lowest and resulted in the lowest final G' value. A large deformation test showed that yogurt with 2.0% buttermilk had the highest yield stress of 94.7 Pa, but yogurt with 4.0% buttermilk had the lowest yield stress of 62.2 Pa (Figure 1b).

The fermentation period for yogurt with 2.0% and 4.0% added buttermilk was shorter than that of other samples. The addition of buttermilk shortened the gelation time and lowered the pH at gelation compared to low‐fat yogurt without the addition of buttermilk. When the concentration of buttermilk was 2.0%, G' value was the highest at pH 4.6 (Table 4), which demonstrated that addition of 2.0% buttermilk improved the rheological properties.

**Table 4:** Rheological properties of low‐fat yogurts fortified with different concentrations of buttermilk powder

	SMP	Addition of buttermilk			
		0.5% BMP	1% BMP	2% BMP	4% BMP
Fermentation period (min)	215.68 ± 4.45^a^	210.32 ± 2.33^a^	213.21 ± 1.32^a^	205.25 ± 2.42^b^	202.32 ± 1.75^b^
Gelation time (min)	135.34 ± 2.35^a^	129.41 ± 2.53^b^	122.29 ± 2.10^c^	126.24 ± 2.42^bd^	127.24 ± 1.56^bd^
pH at gelation	4.90 ± 0.04^a^	4.79 ± 0.03^b^	4.78 ± 0.04^b^	4.80 ± 0.02^b^	4.80 ± 0.02^b^
G′ at pH 4.6 (Pa)	220.46 ± 10.32^c^	196.79 ± 9.32^d^	257.45 ± 8.34^b^	284.35 ± 10.53^a^	136.24 ± 9.43^e^

Note

^a‐d^Means with different letters within the same row are significantly different (*p* < .05).

0.5% BMP, 1% BMP, 2% BMP, and 4% BMP mean low‐fat yogurts with 0%, 0.5%, 1%, 2%, and 4% added buttermilk powder, respectively.

### 3.3 Effect of buttermilk on viscosity and water‐holding capacity of low‐fat yogurts

**Figure 2:**
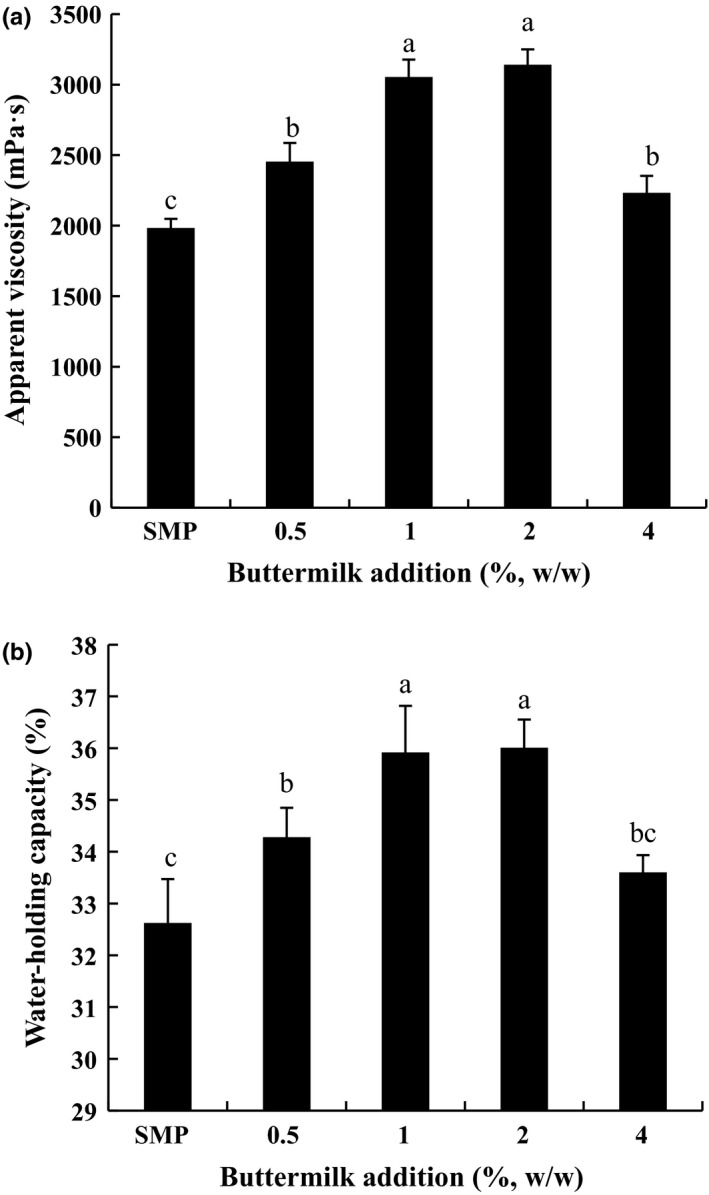
Effect of the addition of buttermilk powder on the (a) viscosity and (b) water‐holding capacity of low‐fat yogurt. SMP, skim milk powder. BMP, buttermilk powder. 0.5% BMP, 1% BMP, 2% BMP, and 4% BMP mean low‐fat yogurts with 0%, 0.5%, 1%, 2%, and 4% added buttermilk powder, respectively

The addition of buttermilk significantly affected the viscosity and water‐holding capacity of low‐fat yogurt (*p < .05*) (Figure 2). Compared with the low‐fat yogurt without buttermilk, addition of 0.5%, 1.0%, 2.0%, and 4.0% buttermilk increased the water‐holding capacity by 5.07%, 10.10%, 10.38%, and 2.99%, and the viscosity by 23.75%, 54.03%, 58.42%, and 12.58%, respectively. Low‐fat yogurt with addition of buttermilk of 1.0% and 2.0% (w/w) obtained the highest water‐holding capacity and viscosity (*p* < .05).

### 3.4 Microstructure of low‐fat yogurts with the addition of buttermilk

**Figure 3:**
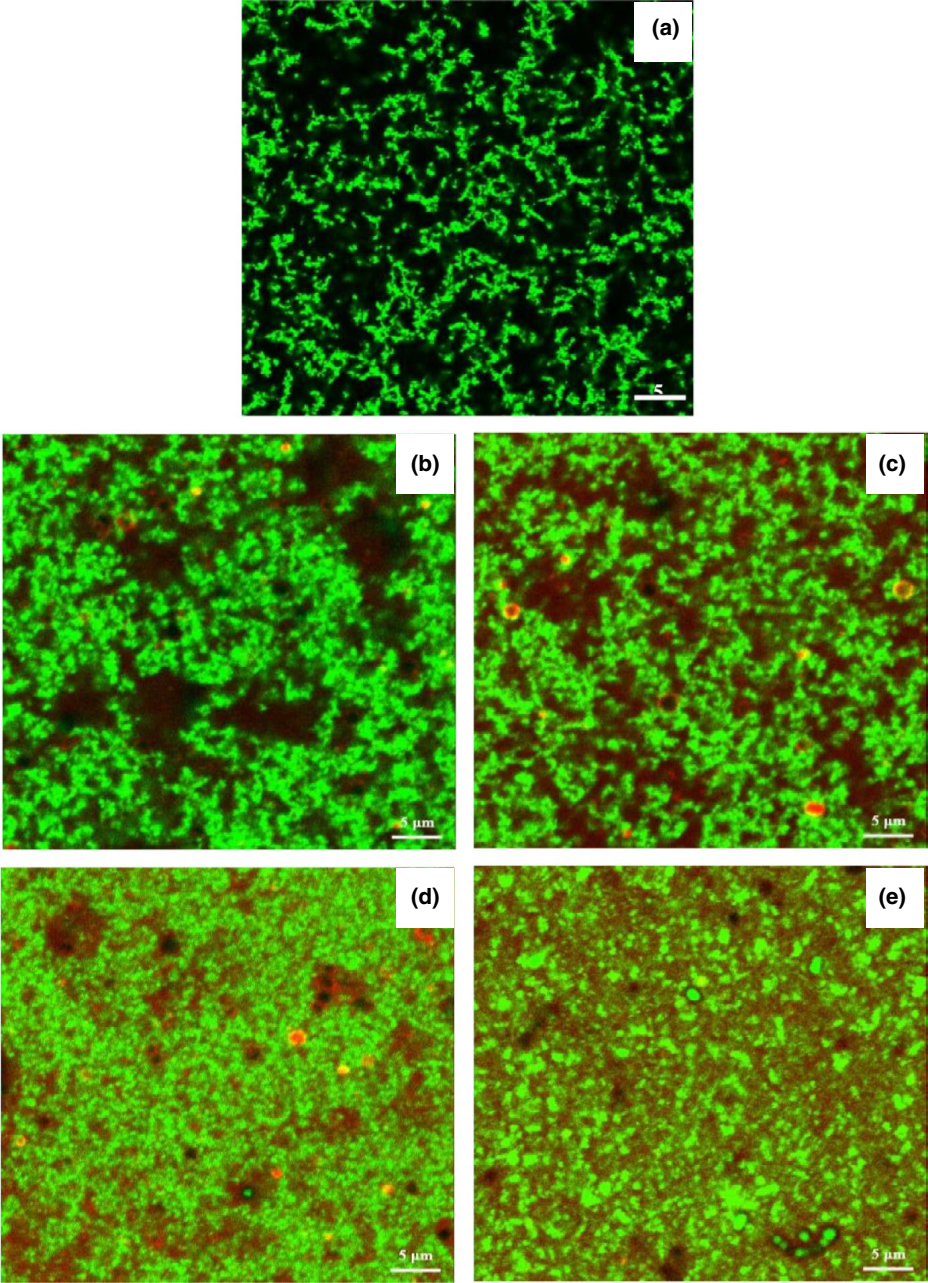
Confocal laser scanning microscopy images of low‐fat yogurts with different concentrations of buttermilk powder (a‐SMP, b‐0.5% BMP, c‐1.0% BMP, d‐2.0% BMP, e‐4.0% BMP). Red color represented the phospholipids labeled with Rh‐DOPE; green color represented the milk proteins labeled with Rhodamine B. SMP, skim milk powder. BMP, buttermilk powder. 0.5% BMP, 1% BMP, 2% BMP, and 4% BMP mean low‐fat yogurts with 0%, 0.5%, 1%, 2%, and 4% added buttermilk powder, respectively

The microstructures of low‐fat yogurt had a more discontinuous structure with larger pores compared to other samples (Figure 3a). As the addition of buttermilk increased, the cross‐linked network became denser and the pores became smaller (Figure 3b, c, d, e). When the added concentration of buttermilk was higher, phospholipids that were labeled with Rh‐DOPE fluorescent dye became more common in the microstructure. When the concentration of buttermilk reached 2.0% (Figure 3d) and 4.0% (Figure 3e), almost all pores in yogurt gels were filled with phospholipids.

### 3.5 Effect of buttermilk on storage properties of low‐fat yogurts

**Figure 4:**
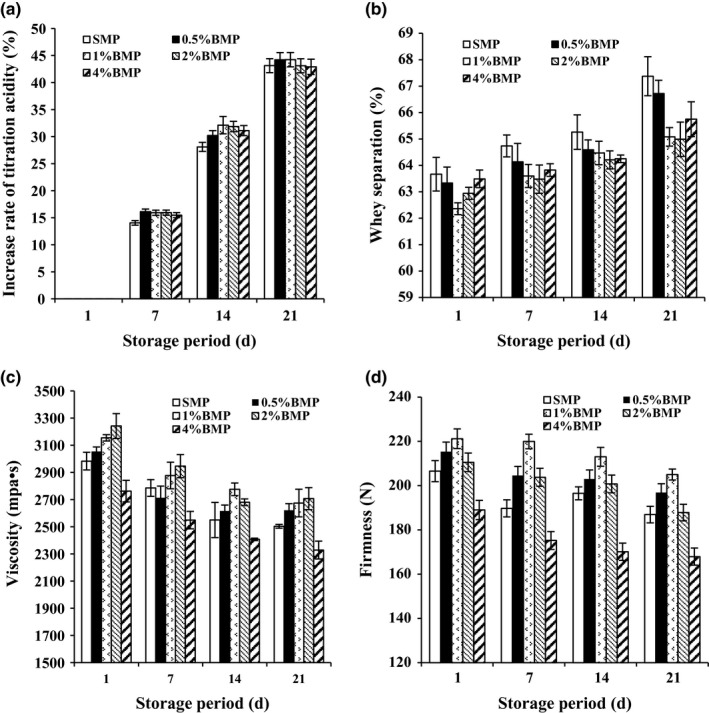
Effect of the addition of buttermilk powder on the (a) titration acidity, (b) viscosity, (c) whey separation, and (d) firmness of low‐fat yogurt during the storage. SMP, skim milk powder. BMP, buttermilk powder. 0.5% BMP, 1% BMP, 2% BMP, and 4% BMP mean low‐fat yogurts with 0%, 0.5%, 1%, 2%, and 4% added buttermilk powder, respectively

Titration acidity of low‐fat yogurt with addition of buttermilk increased during storage period. Compared with low‐fat yogurt without buttermilk, buttermilk increased titration acidity during the first 14 days, but had no significant effect on titration acidity at the end of storage period (Figure 4a). Addition of 1.0% and 2.0% buttermilk significantly decreased the whey separation of low‐fat yogurt during storage (*p* < .05) (Figure 4b). The viscosity and firmness of low‐fat yogurt with addition of buttermilk at 1.0% and 2.0% (w/w) were significantly higher than those of the control without added buttermilk during storage (*p* < .05) (Figure 4c, d). But the addition of buttermilk at 4.0% (w/w) decreased the viscosity and firmness of low‐fat yogurt significantly (*p* < .05) compared with low‐fat yogurt without buttermilk. It demonstrated that buttermilk improved the textural properties of low‐fat yogurt during storage, but not postacidity, and the addition of 1.0% and 2.0% buttermilk were optimum for low‐fat yogurt.

## 4 DISCUSSION

The higher titration acidity and lower pH were observed in yogurt fortified with buttermilk compared with the control without the addition of buttermilk, which contributed to the shorter fermentation time (Table 4). Buttermilk powder may have been beneficial to the proliferation of culture microbes, and it eventually resulted in a higher accumulation of acid. Buttermilk powder might provide low molecular weight peptides or amino acids that were beneficial to the growth of lactic acid (Moe, Porcellato, & Skeie, 2013). In addition, buttermilk was rich in milk fat globule membrane (Spitsberg, 2005). Monosaccharide moieties of glycoconjugates in milk fat globule membrane might also provide an energy source for the starter culture (Moe, Faye, Abrahamsen, Østlie, & Skeie, 2012). Milk fat globule membrane improved the growth and survival of some lactobacilli in low‐fat cheese, and they consequently promoted the ripening of cheddar cheese (Martinovic et al., 2013; Moe et al., 2012). Therefore, buttermilk may possess the ability to improve the growth and metabolism of starter bacteria in yogurts and accelerate the fermentation rate.

Generally, increasing acidification speed and shortening the fermentation period directly have a negative effect on the gel formation of yogurt (Zhang et al., 2010). In the present study, the addition of 1.0%‐2.0% buttermilk led to an increase in final G', yield stress, water‐holding capacity, and viscosity. This was attributed mainly to the components of buttermilk, especially protein and phospholipids, which possess higher emulsifying capacity (Romeih et al., 2012). The MFGM protein in buttermilk interacted with casein and whey protein through disulfide or noncovalent bonds (Lopez, Camier, & Gassi, 2007; Morin, Jimenez‐Flores, & Pouliot, 2007), which enhanced the interaction between milk proteins and improved the textural and rheological properties of low‐fat yogurt. MFGM protein, which effectively reduced moisture loss and retarded staling of wheat bread, confirmed that MFGM protein possesses the ability to improve the textural properties of food (Tang et al., 2016). Phospholipids also have high water‐holding capacity due to their amphiphilic characteristics (Romeih et al., 2014). Phospholipids interacted with whey proteins and β‐casein through electrostatic and hydrophobic interactions (Gallier, Gragson, Jiménez‐Flores, & Everett, 2012; Kasinos et al., 2013). Therefore, Rh‐DOPE‐labeled phospholipid buttermilk may improve the water‐holding capacity and viscosity of low‐fat yogurt (Figure 2). However, Le et al. (2011) found that buttermilk‐supplemented low‐fat yogurts had significantly lower textural properties than the control. The reason for their results might be attributed to the higher content of phospholipids in the buttermilk they used (3.36 g/100 g) compared to the commercial buttermilk in our study (1.25 g/100 g). We also found that addition of buttermilk powder at 4.0% (w/w) that contained 5.0 g/100 g phospholipid made the textural properties of low‐fat yogurt poorer than the control. The amount of phospholipids in 0.5%, 1.0%, and 2.0% buttermilk was 0.63 g/100 g, 1.25 g/100 g, and 2.50 g/100 g, respectively. Although phospholipids are parental molecules, phospholipids have lower hydration than proteins (Dickinson & Chen, 1999), which may decrease water‐holding capacity of yogurt gels. Moreover, excessive phospholipids may have occupied more space and chelated soluble calcium, which ultimately disrupted the gel network (O'Connell & Fox, 2000; Saffon, Britten, & Pouliot, 2011).

The increasing amount of buttermilk in yogurt not only improved the rheological textural properties, but it also decreased the level of whey separation and increased the viscosity and firmness of low‐fat yogurt with 1.0%‐2.0% (w/w) during storage (Figure 4b, c, d). The higher hydration of buttermilk and the interaction between milk protein and MFGM improved the textural and storage properties of low‐fat yogurt. In addition, buttermilk may be a good energy source for *S. thermophiles*. The increased survival of *S. thermophiles* with higher production of exopolysaccharides in yogurt induced the interaction between the milk proteins (Mende, Peter, Bartels, Rohm, & Jaros, 2013). Therefore, the increased survival of *S. thermophiles* may also explain the better textural properties during storage.

## 5 CONCLUSIONS

Addition of 0.5%‐4.0% (w/w) of buttermilk accelerated fermentation rate, shortened the fermentation time, and increased viscosity and water‐holding capacity of low‐fat yogurt. Yogurts with 1.0%‐2.0% (w/w) of buttermilk produced the highest G' and yield stress and had a denser microstructure. In addition, buttermilk also decreased whey separation and improved viscosity and firmness of low‐fat yogurt during storage. Therefore, buttermilk has the potential to improve the rheological and storage properties in fermented dairy products.

## CONFLICT OF INTEREST

All authors declare no conflict of interest.

## ETHICAL STATEMENTS

This study does not involve any human or animal testing.
